# AI Scribe Safety: Measuring What Happens After Signing

**DOI:** 10.2196/103162

**Published:** 2026-08-10

**Authors:** Vera Sorin, Eyal Klang

**Affiliations:** 1 Department of Radiology, Mayo Clinic Hospital, Rochester, MN, United States; 2 Department of Radiology, Beth Israel Deaconess Medical Center, 330 Brookline Avenue, Boston, MA, 02215, United States, 1-617-754-9500

**Keywords:** artificial intelligence, AI, digital scribes, medical scribes, electronic documentation, patient safety, error prevention

## Abstract

Coiera and Fraile-Navarro question whether AI scribes are being evaluated on metrics that truly impact care. While current evaluations focus on the quality of the initial draft, signed clinical notes are dynamic, as their content can be copied, summarized, coded, and re-ingested by downstream AI tools. We argue that safety must be measured downstream, focusing on how small errors in initial documentation can compound across the patient’s electronic health record.

In a recent *JMIR Medical Informatics* editorial, Coiera and Fraile-Navarro ask whether artificial intelligence (AI) scribes are being measured on what matters [1]. We agree with their central point: scribe output shapes what downstream clinicians and systems see and act upon. We build on that point by focusing on what happens to documentation errors after a note is signed and integrated into the health record.

This is far from theoretical. Reddy et al [2] compared notes from 11 ambient AI scribe tools with human notes across 5 standardized primary care visits; they found that human notes scored higher in thoroughness, organization, and usefulness in clinical context . Similarly, a pragmatic pilot study also found omissions, hallucinations, and other errors in AI-generated clinician notes [3]. These flaws can persist beyond the initial draft and propagate after the note is signed.

A signed clinical note is not the end of a visit. Another clinician may read it, a later note may copy it forward, or a referral may summarize it. Orders, billing codes, quality measures, registries, and downstream AI tools may rely on it. Thus, small errors can compound.

For instance, if dyspnea is omitted, the next clinician may not know it was discussed. If a medication dose is wrong, the next medication list may repeat it. If the reason for a plan is missing, a later clinician may follow it without knowing why. In the electronic health record (EHR), an AI-generated omission may be copied, summarized, coded, or used as input for another AI system.

This risk is familiar: EHRs already spread errors through copy-paste, copy-forward, and reused text [4]. Ambient AI scribes add another entry point: one error can become source material for later care.

While clinician review is thus necessary, it cannot catch every flaw. Omissions are hard to detect because the notes do not show omissions. Future studies should follow errors across the lifecycle of the the clinical note: from the visit to the AI draft, signed note, and subsequent notes, referrals, handoffs, orders, patient instructions, registries, and AI summaries. Was the information complete and correct when reused? Was it corrected? Did it affect care? These questions align with established EHR data-quality work [5].

Our goal is not to reject ambient AI scribes, as documentation burden is a critical challenge. However, as Coiera and Fraile-Navarro argue, safety should be measured across the clinical workflow. We maintain that studies should follow the note after signing. The question is not only whether AI scribes produce acceptable first drafts, but whether their errors stay contrained or become part of the patient’s clinical story.

## Abbreviations

AI: artificial intelligence
EHR: electronic health record
